# Efficacy of Single Dose of a Bivalent Vaccine Containing Inactivated Newcastle Disease Virus and Reassortant Highly Pathogenic Avian Influenza H5N1 Virus against Lethal HPAI and NDV Infection in Chickens

**DOI:** 10.1371/journal.pone.0058186

**Published:** 2013-03-01

**Authors:** Dong-Hun Lee, Jae-Keun Park, Jung-Hoon Kwon, Seong-Su Yuk, Tseren-Ochir Erdene-Ochir, Yo-Han Jang, Baik-Lin Seong, Joong-Bok Lee, Seung-Yong Park, In-Soo Choi, Chang-Seon Song

**Affiliations:** 1 Avian Disease Laboratory, College of Veterinary Medicine, Konkuk University, Seoul, Republic of Korea; 2 Department of Biotechnology, College of Life Science and Biotechnology, Yonsei University, Seoul, Republic of Korea; The University of Hong Kong, Hong Kong

## Abstract

Highly pathogenic avian influenza (HPAI) and Newcastle disease (ND) are 2 devastating diseases of poultry, which cause great economic losses to the poultry industry. In the present study, we developed a bivalent vaccine containing antigens of inactivated ND and reassortant HPAI H5N1 viruses as a candidate poultry vaccine, and we evaluated its immunogenicity and protective efficacy in specific pathogen-free chickens. The 6∶2 reassortant H5N1 vaccine strain containing the surface genes of the A/Chicken/Korea/ES/2003(H5N1) virus was successfully generated by reverse genetics. A polybasic cleavage site of the hemagglutinin segment was replaced by a monobasic cleavage site. We characterized the reverse genetics-derived reassortant HPAI H5N1 clade 2.5 vaccine strain by evaluating its growth kinetics in eggs, minimum effective dose in chickens, and cross-clade immunogenicity against HPAI clade 1 and 2. The bivalent vaccine was prepared by emulsifying inactivated ND (La Sota strain) and reassortant HPAI viruses with Montanide ISA 70 adjuvant. A single immunization with this vaccine induced high levels of hemagglutination-inhibiting antibody titers and protected chickens against a lethal challenge with the wild-type HPAI and ND viruses. Our results demonstrate that the bivalent, inactivated vaccine developed in this study is a promising approach for the control of both HPAI H5N1 and ND viral infections.

## Introduction

Avian influenza virus (AIV) is a member of the Influenza virus A genus belonging to the *Orthomyxoviridae* family [1]. AIV infection can cause various disease symptoms in chickens, ranging from asymptomatic infection to respiratory disease, and reduced egg production to severe systemic diseases with near 100% mortality rates. Genetic features and/or severity of the disease in poultry determine whether the infection is classified as low pathogenic avian influenza (LPAI) or high pathogenic avian influenza (HPAI). The hemagglutinin (HA) gene is a key determinant of virulence in poultry species. Posttranslational cleavage of the HA precursor molecule by Furin-like cellular enzymes is essential for efficient virus spreading, leading to systemic infection and high mortality [2]. The HPAI subtype H5N1, defined as a ‘‘notifiable’’ disease by the World Organisation for Animal Health (OIE), has caused fatal infections in poultry with severe economic impact worldwide [3]. In particular, the Asian lineage of HPAI H5N1 has become widespread across continents, including Eurasia and Africa, and the spread of the virus in poultry in Southeast Asia has become endemic since it was first identified in China in 1996 [4]. Furthermore, to date, HPAI H5N1 has been confirmed in more than 500 human cases with approximately 60% fatality rate, thus raising global public health concerns about its pandemic potential [5].

Although vaccination of poultry species against HPAI has been discouraged in the past and is still regarded as a controversial topic, it is now recommended as one of the HPAI control strategies [6]. Production of a safe and high-yield HPAI H5N1 vaccine strain is challenging for vaccine manufacturers; therefore, the reverse genetics system, which uses a high-growth backbone virus, offers a solution for the generation of a high-yield, avirulent HPAI vaccine strain for vaccination of poultry species [7]–[10].

Newcastle disease (ND) caused by avian paramyxovirus serotype 1 (APMV-1), also known as Newcastle disease virus (NDV), is considered as one of the most devastating poultry infections, owing to its worldwide distribution and economical threat. According to disease severity in chickens, NDVs have been categorized into lentogenic, mesogenic, and velogenic strains [11]. Velogenic strains can cause sudden death of fully susceptible chickens. Typically, disease signs such as depression, prostration, diarrhea, and nervous signs may occur, and flock mortality may reach 100%. Highly virulent velogenic NDV genotype VII viruses have been previously isolated in Asian countries, including Korea [12], China [13], Japan [14], and Taiwan [15]. In addition, in Europe, 230 ND outbreaks have been reported in 13 of the 27 EU member states in 2005–2009 [16]. Currently, in order to keep the spread of ND under control, many countries worldwide maintain a stringent vaccination policy. A classic live vaccine based on the lentogenic LaSota strain, is routinely applied to chickens and has been proved to induce high levels of immunogenicity and protective efficacy against lethal velogenic strains [17], [18].

In the present study, we characterized a reverse genetics-derived reassortant HPAI H5N1 clade 2.5 virus by evaluating its growth kinetics in eggs, minimum effective dose in chickens, and cross-clade immunogenicity against HPAI clade 1 and 2. Furthermore, we developed a bivalent vaccine containing the inactivated NDV LaSota strain and reassortant H5N1 virus for vaccination in poultry. We also evaluated its immunogenicity and protective efficacy against lethal HPAI H5N1 and virulent NDV genotype VII virus infection by using specific pathogen-free (SPF) chickens.

## Materials and Methods

### Ethics Statement

All animal procedures performed in this study (permit number: KU12025) were reviewed, approved, and supervised by the Institutional Animal Care and Use Committee of Konkuk University.

### Generation of a Reassortant H5N1 Virus

The reassortant H5N1 vaccine was generated by a reverse genetics approach, as previously described [19]. Briefly, HA and neuraminidase (NA) genes of A/Chicken/Korea/ES/2003 (H5N1) virus (clade 2.5) were synthesized and cloned into the pHW2000 vector. To ensure safety of the reassortant viruses, a polybasic cleavage site (PQRESRRKKRG) of the HA segment was replaced by a monobasic cleavage site (PQREKRG). pHW2000-HA and pHW2000-NA plasmids were mixed with 6 internal plasmids of the X-31ca virus and transfected into 293T cells.

### Characterization of the Reassortant H5N1 Virus

We evaluated the reverse genetics-derived reassortant H5N1 virus strain’s growth kinetics in eggs, minimum effective dose required in chickens, and cross-clade immunogenicity against HPAI clade 1 and 2 viruses. To determine growth kinetics of the reassortant H5N1 virus in SPF embryonated chicken eggs, escalating doses (2.3, 3.3, and 4.3 log EID_50_/egg) of the reassortant H5N1 virus were inoculated into the allantoic cavity of 10-day-old SPF embryonated chicken eggs. After 24, 48, and 72 hours of incubation, the eggs were chilled and allantoic fluids were harvested in axenic and tested for hemagglutination activity. For 72 hour-incubated allantoic fluids, virus titers were determined using SPF embryonated chicken eggs and calculated by the Reed–Muench method [20]. In addition, the minimum effective dose and cross-clade immunogenicity against HPAI clade 1 and 2 viruses were analyzed using SPF chickens.

### Vaccine and Viruses

To determine the minimum effective dose of the reassortant H5N1 virus, monovalent H5N1 vaccines were prepared by emulsifying escalating doses (5.0, 6.0, 7.0 and 8.0 log EID_50_/dose) of the inactivated reassortant H5N1 virus with Montanide ISA 70 (SEPPIC, France) at a ratio of 30∶70 (v/v).

A bivalent vaccine was prepared by emulsifying inactivated reassortant H5N1 (10^7.5^ EID_50_/dose) and lentogenic NDV LaSota (10^6.0^ EID_50_/dose) strains with Montanide ISA 70 at a ratio of 30∶70 (v/v). To evaluate the vaccine efficacy, chickens were challenged intranasally with 10^5.0^ EID_50_ of wild-type HPAI H5N1 virus (A/Chicken/Korea/ES/2003) or intramuscularly with 10^5.5^ EID_50_ of the virulent NDV genotype VII virus (Kr-005/00). The A/Chicken/Korea/ES/2003(H5N1) virus was provided by the Animal, Plant and Fisheries Quarantine and Inspection Agency, Korea.

### Animals and Experimental Design

The minimum effective dose of the reassortant H5N1 virus was determined using SPF chickens (Namduck Sanitec, Korea). Four groups of chickens (n = 10) were intramuscularly immunized with 0.5 ml of escalating doses (5.0, 6.0, 7.0, and 8.0 log EID_50_/dose) of the monovalent H5N1 vaccine. Hemagglutination-inhibition (HI) antibody titers against the homologous antigen were determined 3 weeks after vaccination.

For evaluating the immunogenicity and protective efficacy of the bivalent vaccine, fifty 6-week-old SPF chickens were divided into 4 groups. Twenty-five chickens were immunized intramuscularly with 0.5 ml of the vaccine. As a non-vaccinated control group, another 25 chickens were injected with an emulsified mixture of the adjuvant and distilled water. Serum samples were collected 3 weeks after vaccination and used to determine antibody titers.

Three weeks after a single dose of the vaccine, chickens were challenged intranasally with the wild-type HPAI virus under biosafety level 3 conditions. We observed mortality and clinical signs daily for 10 days post-challenge (dpc). In addition, viral shedding was quantified at 5 dpc by real-time reverse transcriptase polymerase chain reaction (rRT-PCR).

Similarly, 3 weeks after a single dose of the vaccine was administered, chickens were challenged intramuscularly with the virulent NDV virus under biosafety level 2-enhanced conditions. Mortality and clinical manifestations (depression, diarrhea, and neurologic signs) were observed daily for 14 dpc.

### Virus Quantification

To determine the HPAI virus shedding, oropharyngeal and cloacal swab samples were collected at 5 dpc and suspended in 1 ml of phosphate-buffered saline supplemented with gentamycin (400 µg/ml). Of this suspension, 200 µl was used for RNA extraction with RNeasy Mini Kit (QIAGEN) according to the manufacturer’s instruction. RNA concentration was quantified by the cycle threshold (Ct) method using M gene-based rRT-PCR, as previously described [21]. For conversion of Ct values to infectious units, known titers of A/Chicken/Korea/ES/2003(H5N1) virus from egg allantoic fluid (measured in EID_50_) were 10-fold serially diluted and analyzed by rRT-PCR as described above. For generation of a standard curve, Ct values of each viral dilution were plotted against viral titers. The resulting standard curve had a high correlation coefficient (r^2^>0.99), and it was used to convert Ct values to EID_50_.

### Serology

In order to determine the immunogenicity of the vaccines, serum samples were collected prior to vaccination and at 3 weeks after vaccination for HI test. HI tests were performed using formalin-inactivated homologous and heterologous antigens as previously described [3]. To determine cross-clade immunogenicity of the reassortant H5N1 vaccine, reverse genetics-derived influenza A/Indonesia/5/2005 (clade 2.1) and A/Vietnam/1194/2004 (clade 1) viruses were formalin-inactivated and used as heterologous antigens. The 6∶2 reassortant H5N1 strains containing the HA and NA genes of the clade 1 and 2.1 were kindly provided by Dr. Baik-Lin Seong, Yonsei University, Korea.

### Statistical Analysis

Analysis of variance along with a Tukey–Kramer post-hoc test was performed for serum HI antibody titers. Results with P values <0.05 were considered statistically significant.

## Results and Discussion

**Figure 1:**
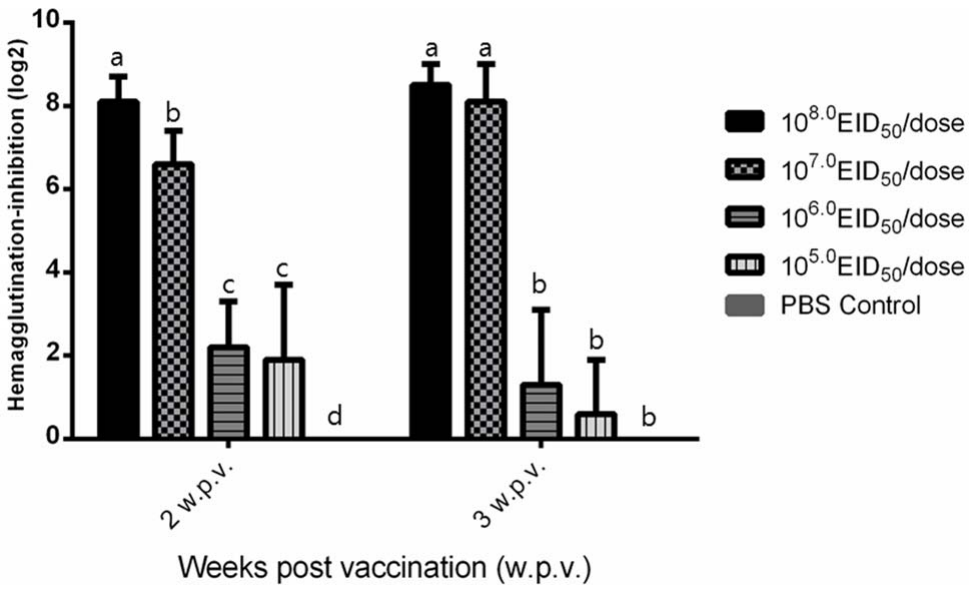
Immunogenicity of the reassortant H5N1 virus in specific pathogen-free (SPF) chickens. Vaccines were prepared by emulsifying escalating doses (5.0, 6.0, 7.0, and 8.0 log EID_50_/ml) of the inactivated reassortant H5N1 virus with Montanide ISA 70 at a ratio of 30∶70 (v/v). Groups of SPF chickens were intramuscularly immunized with the vaccine. HI titers against the homologous antigen were determined at 2 and 3 weeks after vaccination. In the graph, values represented with same superscript letters for a particular week are not significantly different (p<0.05).

The 6∶2 reassortant H5N1 vaccine strain containing the HA and NA genes of the A/Chicken/Korea/ES/2003(H5N1) virus was successfully generated by reverse genetics. A polybasic cleavage site of the HA segment was replaced by a monobasic cleavage site. According to a pathogenicity test using chickens and eggs, the reassortant H5N1 vaccine strain did not induce any mortality in 10-day-old SPF chicken embryos and 1-day-old SPF chicks (data not shown). For optimization of the vaccine production process, we analyzed growth kinetics of the reassortant H5N1 virus in SPF embryonated chicken eggs and its minimum effective dose in chickens. In eggs inoculated with 2.3 log EID_50_ of reassortant virus, virus replication was not detected at 24 hours post-inoculation (pi), but virus titers were the highest at 72 hours pi (Table 1). We compared the immunogenicity of the reassortant H5N1 virus in SPF chickens with different antigen contents. As shown in Fig. 1, 2 weeks after vaccination 8.0 log EID_50_ dose of the oil-emulsified vaccine induced significantly higher HI antibody titers in chickens than other doses. Chickens belonging to the 7.0 log EID_50_/dose group showed higher HI antibody titers than those belonging to the 6.0 and 5.0 EID_50_/dose groups. Three weeks after vaccination, chickens belonging to the 7.0 and 8.0 log EID_50_/dose groups showed significantly higher HI antibody titers than other groups. Therefore, the vaccine based on the inactivated reassortant H5N1 strain induced high antibody titers at a dose of 7.0–8.0 log EID_50_.

**Table 1:** Growth kinetics of the reassortant H5N1 virus in SPF embryonated chicken eggs.

Inoculum^A^ (logEID_50_/egg)	Quantities of propagated virus in eggs		
	24 h post inoculation	48 h post inoculation	72 h post inoculation
4.3	4.6±0.8 HAU	6.9±0.6 HAU	7.2±0.6 HAU
			7.8 log EID_50_/ml
3.3	1.6±1.7 HAU	7.3±0.5 HAU	7.5±0.5 HAU
			7.2 log EID_50_/ml
2.3	0±0.0 HAU	7.6±0.5 HAU	7.8±0.6 HAU
			8.3 log EID_50_/ml

A 0.1 ml of each dilution was inoculated into the allantoic cavity of 10-day-old SPF embryonated chicken eggs.

HAU, log2 hemagglutination unit.

To evaluate the immunogenicity of the bivalent vaccine, HI antibody titers were determined 3 weeks after a single immunization. Throughout the experiment, no signs of adverse effects (e.g., unusual local or systemic response) were observed in any of the vaccinated chickens. As shown in Tables 2 and 3, the vaccine significantly increased HI antibody titers against homologous or heterologous antigens in chickens. The HI titers against the homologous antigens (clade 2.5) were higher than those against the heterologous HI antigens (clades 1 and 2.1), and among the heterologous antigens HI titers against clade 2.1 were at higher levels than those against clade 1 viruses, reflecting the antigenic similarity. For NDV, mean HI antibody titers against the homologous antigens was 2^7.5^ in vaccinated chickens (Table 4). However, no HI antibody responses were detected in the non-vaccinated group that received the emulsified mixture of the ISA 70 adjuvant with distilled water. These results suggest that the bivalent vaccine developed in this study induces anti-H5N1 and anti-NDV functional antibody responses in chickens without any adverse effects.

**Table 2:** Immunogenicity of the reassortant H5N1 virus against homologous and heterologous clade viruses.

Vaccinated chicken	Hemagglutination-inhibition titer (log2)		
	Homologous clade	Heterologous clade	
	Clade 2.5	Clade 2.1	Clade 1
No. 1	8	5	4
No. 2	8	7	4
No. 3	8	6	5
No. 4	6	4	2
No. 5	8	6	4
No. 6	7	4	2
No. 7	8	7	4
No. 8	7	5	2
Mean titer ± S.D.	7.5±0.8	5.5±1.2	3.4±1.1

**Table 3:** Protective efficacy of the bivalent inactivated vaccine against lethal HPAI H5N1 virus infection.

Group^A^	HI titer^B^ (log2)	Mortality (MDT)	Clinical sign^C^	Virus replication^D^ (logEID_50_/ml)	
				Oropharynx	Cloaca
Vaccinated	9.0±0.7	0/10	0/10	1/10 (0.19)	1/10 (0.12)
Non-Vaccinated	0	10/10 (2.8)	10/10	N/A	N/A

A SPF chickens were challenged intranasally with 100 µl of 10^5.0^ EID_50_ of homologous H5N1 virus 3 weeks post-vaccination.

B Hemagglutination-inhibition titers against the homologous antigen were determined 3 weeks after vaccination using chicken erythrocytes.

C Depression was observed in non-vaccinated chickens before death.

D Virus replication was determined at day 5 post-infection. Log EID_50_ equivalents were determined by quantitative real-time reverse transcriptase polymerase chain reaction.

MDT, mean death time in days.

N/A, not applicable.

**Table 4:** Protective efficacy of the bivalent inactivated vaccine against lethal Newcastle disease virus infection.

Group^A^	HI titer^B^ (log2)	Mortality (MDT)	Clinical signs^C^
Vaccinated	7.5±0.9	0/15	0/15
Non-Vaccinated	0	15/15 (4.0)	15/15

A SPF chickens were challenged intramuscularly with 100 µl of 10^5.5^ EID_50_ of the virulent NDV virus 3 weeks post-vaccination.

B Hemagglutination-inhibition titers against the homologous antigen were determined 3 weeks after vaccination using chicken erythrocytes.

C Depression was observed in non-vaccinated chickens before death.

MDT, mean death time in days.

To examine the protective efficacy of the vaccine, chickens were infected with a high dose of HPAI or NDV 3 weeks after a single immunization dose. Non-vaccinated chickens showed severe clinical signs and 100% mortality after challenge with HPAI (mean death time [MDT] = 2.8 day) or NDV (MDT = 4.0 day) (Tables 3 and 4). At the same time, 100% of the vaccinated chickens were protected from mortality, showed no clinical signs of HPAI infection, and only 1 of 10 chickens was positive for viral replication in the oropharynx and cloaca. For NDV, all vaccinated chickens survived lethal virulent NDV infection without any clinical signs.

Greatly advanced molecular techniques and availability of information on the influenza A virus genome create new opportunities for development of novel vaccine technologies. For a cost-effective and highly immunogenic HPAI vaccine, a seed virus strain should have high growth rates in culture and appropriate antigenicity against current wild-type viruses. The reverse genetics system has been widely used in influenza vaccine development for improvement of the ability of vaccine strains to replicate in eggs [22], [23]. In addition, a reverse genetics-derived strain used in the vaccine against HPAI has potential advantages in terms of safety and antigenic similarity, because the pathogenic motif can be easily removed and genetically attenuated vaccine strains can be produced without using high-level biocontainment facilities. Finally, using the reverse genetics system, a reassortant vaccine seed strain consisting of 6 internal genes of a high-growth donor strain and 2 surface genes from a wild-type virus can be generated as an alternative to the classical multiple passage procedure, thus allowing expression of the wild-type virus epitopes.

Frequent economic losses incurred by HPAI and ND infections have raised serious concerns for the poultry industry. Previously, several bivalent vaccines against NDV and HPAI H5 viruses were developed using a recombinant NDV vaccine strain as a vector to express the HA protein from an HPAI H5N1 virus [24], [25]. Although these chimeric vaccines provided clinical protection and reduced wild-type virus shedding, there are safety issues associated with the use of NDV live attenuated virus and possible genetic exchange [26]. The bivalent vaccine against NDV and HPAI H5N1 developed in the present study induced high titers of both HPAI H5 and NDV-specific antibodies and afforded complete protection against lethal challenge with NDV and HPAI H5N1. Most importantly, since the vaccine strains are inactivated and genetically stable, there is no danger of shaping NDV evolution by homologous recombination with the wild-type virus, which resolves the above-mentioned problems of vaccine safety.

In conclusion, the reverse genetics-derived reassortant H5N1 virus strain was avirulent, immunogenic, and exhibited high growth yield properties in eggs. The bivalent vaccine against NDV and HPAI H5N1 developed in the present study was also safe, and immunogenic, and it protected SPF chickens from lethal HPAI and NDV infections in terms of protection from mortality. The results obtained in this study demonstrate that the use of the bivalent vaccine against HPAI and NDV in poultry species is a promising strategy for controlling both HPAI H5N1 and virulent NDV infections.

## References

[1] Webster RG, Bean WJ, Gorman OT, Chambers TM, Kawaoka Y (1992) Evolution and ecology of influenza A viruses. Microbiol Rev 56: 152–179. 1579108 10.1128/mr.56.1.152-179.1992 PMC372859

[2] Rott R (1992) The pathogenic determinant of influenza virus. Vet Microbiol 33: 303–310. 1481363 10.1016/0378-1135(92)90058-2

[3] World-Organization-for-Animal-Health (2009) OIE Terrestrial Manual, CHAPTER 2.3.4, AVIAN INFLUENZA.

[4] Webster RG, Hulse-Post DJ, Sturm-Ramirez KM, Guan Y, Peiris M, et al (2007) Changing epidemiology and ecology of highly pathogenic avian H5N1 influenza viruses. Avian Dis 51: 269–272. 17494564 10.1637/7641-050206R.1

[5] World-Health-Organization (2011) Cumulative number of confirmed human cases of avian influenza A(H5N1) reported to WHO.

[6] Butler D (2005) Vaccination will work better than culling, say bird flu experts. Nature 434: 810. 10.1038/4344810a 15829925

[7] Tian G, Zeng X, Li Y, Shi J, Chen H (2010) Protective efficacy of the H5 inactivated vaccine against different highly pathogenic H5N1 avian influenza viruses isolated in China and Vietnam. Avian Dis 54: 287–289. 20521646 10.1637/8707-031709-ResNote.1

[8] Shi H, Liu XF, Zhang X, Chen S, Sun L, et al (2007) Generation of an attenuated H5N1 avian influenza virus vaccine with all eight genes from avian viruses. Vaccine 25: 7379–7384. 17870216 10.1016/j.vaccine.2007.08.011

[9] Tian G, Zhang S, Li Y, Bu Z, Liu P, et al (2005) Protective efficacy in chickens, geese and ducks of an H5N1-inactivated vaccine developed by reverse genetics. Virology 341: 153–162. 16084554 10.1016/j.virol.2005.07.011

[10] Webster RG, Webby RJ, Hoffmann E, Rodenberg J, Kumar M, et al (2006) The immunogenicity and efficacy against H5N1 challenge of reverse genetics-derived H5N3 influenza vaccine in ducks and chickens. Virology 351: 303–311. 16690097 10.1016/j.virol.2006.01.044

[11] Alexander DJ (1997) Newcastle disease and other avian paramyxoviridae infections. In: Calnek BW, Barnes HJ, Beared CW, McDougald LR, Saif YM, editors. Diseases of Poultry. Iowa: Iowa State University Press. 541–569.

[12] Lee YJ, Sung HW, Choi JG, Kim JH, Song CS (2004) Molecular epidemiology of Newcastle disease viruses isolated in South Korea using sequencing of the fusion protein cleavage site region and phylogenetic relationships. Avian Pathol 33: 482–491. 15545028 10.1080/03079450400003700

[13] Liang R, Cao DJ, Li JQ, Chen J, Guo X, et al (2002) Newcastle disease outbreaks in western China were caused by the genotypes VIIa and VIII. Vet Microbiol 87: 193–203. 12052330 10.1016/s0378-1135(02)00050-0

[14] Mase M, Imai K, Sanada Y, Sanada N, Yuasa N, et al (2002) Phylogenetic analysis of Newcastle disease virus genotypes isolated in Japan. J Clin Microbiol 40: 3826–3830. 12354891 10.1128/JCM.40.10.3826-3830.2002 PMC130906

[15] Ke GM, Liu HJ, Lin MY, Chen JH, Tsai SS, et al (2001) Molecular characterization of Newcastle disease viruses isolated from recent outbreaks in Taiwan. J Virol Methods 97: 1–11. 11483212 10.1016/s0166-0934(01)00296-8

[16] OIE (2010) World Animal Health Information Database (WAHID) Interface.

[17] Liu XF, Wan HQ, Ni XX, Wu YT, Liu WB (2003) Pathotypical and genotypical characterization of strains of Newcastle disease virus isolated from outbreaks in chicken and goose flocks in some regions of China during 1985–2001. Arch Virol 148: 1387–1403. 12827467 10.1007/s00705-003-0014-z

[18] Jeon WJ, Lee EK, Lee YJ, Jeong OM, Kim YJ, et al (2008) Protective efficacy of commercial inactivated Newcastle disease virus vaccines in chickens against a recent Korean epizootic strain. J Vet Sci 9: 295–300. 18716450 10.4142/jvs.2008.9.3.295 PMC2811842

[19] Jung EJ, Lee KH, Seong BL (2010) Reverse genetic platform for inactivated and live-attenuated influenza vaccine. Exp Mol Med 42: 116–121. 20054235 10.3858/emm.2010.42.2.013 PMC2827828

[20] Reed LJ, Muench H (1938) A simple method of estimating fifty percent endpoint. The American Journal of Hygiene 27: 493–497.

[21] Spackman E, Senne DA, Bulaga LL, Myers TJ, Perdue ML, et al (2003) Development of real-time RT-PCR for the detection of avian influenza virus. Avian Dis 47: 1079–1082. 14575115 10.1637/0005-2086-47.s3.1079

[22] Engelhardt OG (2012) Many ways to make an influenza virus - review of influenza virus reverse genetics methods. Influenza Other Respi Viruses. 10.1111/j.1750-2659.2012.00392.x PMC5779834 22712782

[23] Neumann G, Ozawa M, Kawaoka Y (2012) Reverse genetics of influenza viruses. Methods Mol Biol 865: 193–206. 22528161 10.1007/978-1-61779-621-0_12

[24] Ge J, Deng G, Wen Z, Tian G, Wang Y, et al (2007) Newcastle disease virus-based live attenuated vaccine completely protects chickens and mice from lethal challenge of homologous and heterologous H5N1 avian influenza viruses. J Virol 81: 150–158. 17050610 10.1128/JVI.01514-06 PMC1797253

[25] Veits J, Wiesner D, Fuchs W, Hoffmann B, Granzow H, et al (2006) Newcastle disease virus expressing H5 hemagglutinin gene protects chickens against Newcastle disease and avian influenza. Proc Natl Acad Sci U S A 103: 8197–8202. 16717197 10.1073/pnas.0602461103 PMC1472452

[26] Han GZ, Liu XP, Li SS (2008) Caution about newcastle disease virus-based live attenuated vaccine. J Virol 82: 6782–6782. 18544791 10.1128/JVI.00370-08 PMC2447105

